# The use of autologous platelet-rich plasma in embryo culture to obtain high-quality embryos

**DOI:** 10.1038/s41598-025-07688-x

**Published:** 2025-07-18

**Authors:** Yeo Jin Rhee, Jae Kyun Park, So-Yeon Ahn, Soyoung Bang, Jung Hoon Kim, Min Kyoung Kim, Woo Sik Lee

**Affiliations:** 1 Department of Obstetrics and Gynecology, Fertility Center of CHA Gangnam Medical Center, CHA University School of Medicine, Seoul, Republic of Korea; 2 Seoul Fertility Clinic, Seoul, Republic of Korea

**Keywords:** Autologous platelet-rich plasma (PRP), Embryo culture medium, Embryo development, Embryo quality, Cytokines, In vitro fertilization outcome, Endocrine reproductive disorders, Infertility

## Abstract

Transferring high-quality embryos is crucial for in vitro fertilization success, as they lead to better clinical pregnancy and live birth outcomes. Researchers have investigated adding supplements to culture media to enhance embryo quality because culture conditions greatly affect embryo quality and development. This study assessed whether adding autologous platelet-rich plasma containing growth factors and cytokines to the culture media could produce more usable and high-grade embryos. This retrospective study analyzed 175 in vitro fertilization cycles from 123 women with poor embryo development or no usable embryos in previous cycles. 5% platelet-rich plasma solution was added to the cleavage-stage culture medium, and embryos were incubated for 48 h. Embryo development rates, stage-specific usable embryos, and high-quality embryo proportions were measured. Cytokine analysis was performed to compare the platelet-rich plasma samples from patients with high- and low-quality embryos. Adding autologous platelet-rich plasma significantly improved embryo development outcomes, with higher usable embryo rates compared to untreated groups. Platelet-rich plasma enhanced outcomes at the blastocyst stage and improved high-quality embryo rates at the morula and blastocyst stages. Both POSEIDON groups II and IV showed significantly better usable embryo ratios with platelet-rich plasma. Significant differences in cytokine expression levels were observed between platelet-rich plasma samples from patients with high- and low-quality embryo samples, with notable variations in Flt-3 ligand, interleukin-23, monocyte chemoattractant protein-3, and urokinase plasminogen activator receptor. The limitations of this study are retrospective design without a placebo control group and its small sample size. Nevertheless, our findings are valuable for patients with poor prognoses, showing improved embryo development outcomes. This offers opportunities for older patients with multiple failed in vitro fertilization attempts.

## Introduction

Since the first child born through in vitro fertilization (IVF), assisted reproductive technology (ART) has provided many infertile couples with opportunities for conception and childbirth. Different techniques have been introduced to augment pregnancy rates, live birth rates, and other ART outcomes. One of these techniques involves the use of platelet-rich plasma (PRP), i.e., an autologous concentration of platelets in the plasma^1^. It contains numerous cytokines and growth factors that can exert a regenerative effect and induce cell proliferation in injured tissues. PRP therapy has been used in animal and human trials. Particularly in women with endometrium- and ovary-associated infertility, PRP has been administered via intrauterine and intraovarian injections to improve ART outcomes^2–5^. Serdarogullari et al. reviewed studies focusing on increased endometrial thickness and ovarian rejuvenation to evaluate the role of PRP in enhancing female infertility. Although most studies had differences in their design with small sample sizes, the majority of studies using PRP to treat thin endometrium demonstrated an improvement in implantation rates. Additionally, studies involving patients with recurrent implantation failure also showed that PRP treatment enhanced pregnancy rates^5^.

Over the past few years, significant advancements have been observed in embryo culture. Previous studies have highlighted the importance of culture conditions in influencing embryo quality and development^6^. Several studies of animal models have demonstrated that adding various supplements to culture media improved oocyte maturation and embryo quality^7–10^. A recent study demonstrated improved viability and growth in isolated human follicles in vitro when the culture media was added with PRP compared with other serum supplements^11^. However, to the best of our knowledge, no studies have investigated whether the addition of PRP to the embryo culture media promotes human embryo development or quality.

In this study, we investigated whether the addition of autologous PRP to the culture media during embryo culture could positively affect human embryo development, quality, and embryo freezing rate. In addition, we explored whether cytokine expressions differ between the platelet-rich plasma samples from patients with high- and low-quality embryos using a cytokine array kit.

## Materials and methods

### Ethical approval

This study was approved by the ethics committee of the Institutional Review Board of CHA Gangnam Medical Center (IRB approval no. GCI 2023–02-006). Given the retrospective design of the study and its reliance on medical records, the need for obtaining participant consent was waived by the approval committee. However, the study was conducted in strict adherence to relevant privacy regulations and guidelines.

### Patient eligibility criteria

This retrospective study included patients with infertility who underwent consecutive IVF treatments at a single center, Fertility Center of CHA Gangnam Medical Center, between August 2021 and February 2024. A total of 175 cycles were analyzed, comprising 123 patients. The inclusion criteria were as follows: previous IVF cycles that exhibited cleavage-stage embryo development even after extended culture, low-quality blastocysts, and no usable embryos.

### Patient stratification

In previous studies, female patients with low prognosis were classified into four subgroups based on the POSEIDON criteria^12^. To clarify the effect of PRP on embryo development in poor responders, participants were classified according to the POSEIDON criteria, and the differences between each group were analyzed. All women underwent ovarian reserve assessment within 1–3 months before starting ovarian stimulation. Ovarian reserve evaluation (anti-Müllerian hormone [AMH] and antral follicle count [AFC]) was performed on menstrual cycle day 2 or 3 before the start of ovarian stimulation. In this study, subgroups were classified based on the combined maternal age and AMH (excluding AFC) and categorized into four subgroups: group 1, maternal age < 35 years and AMH ≥ 1.2 ng/mL; group 2, maternal age ≥ 35 years and AMH ≥ 1.2 ng/mL; group 3, women aged < 35 years and AMH < 1.2 ng/mL, and group 4, women aged ≥ 35 years and AMH < 1.2 ng/mL.

### Autologous PRP preparation

**Fig. 1:**
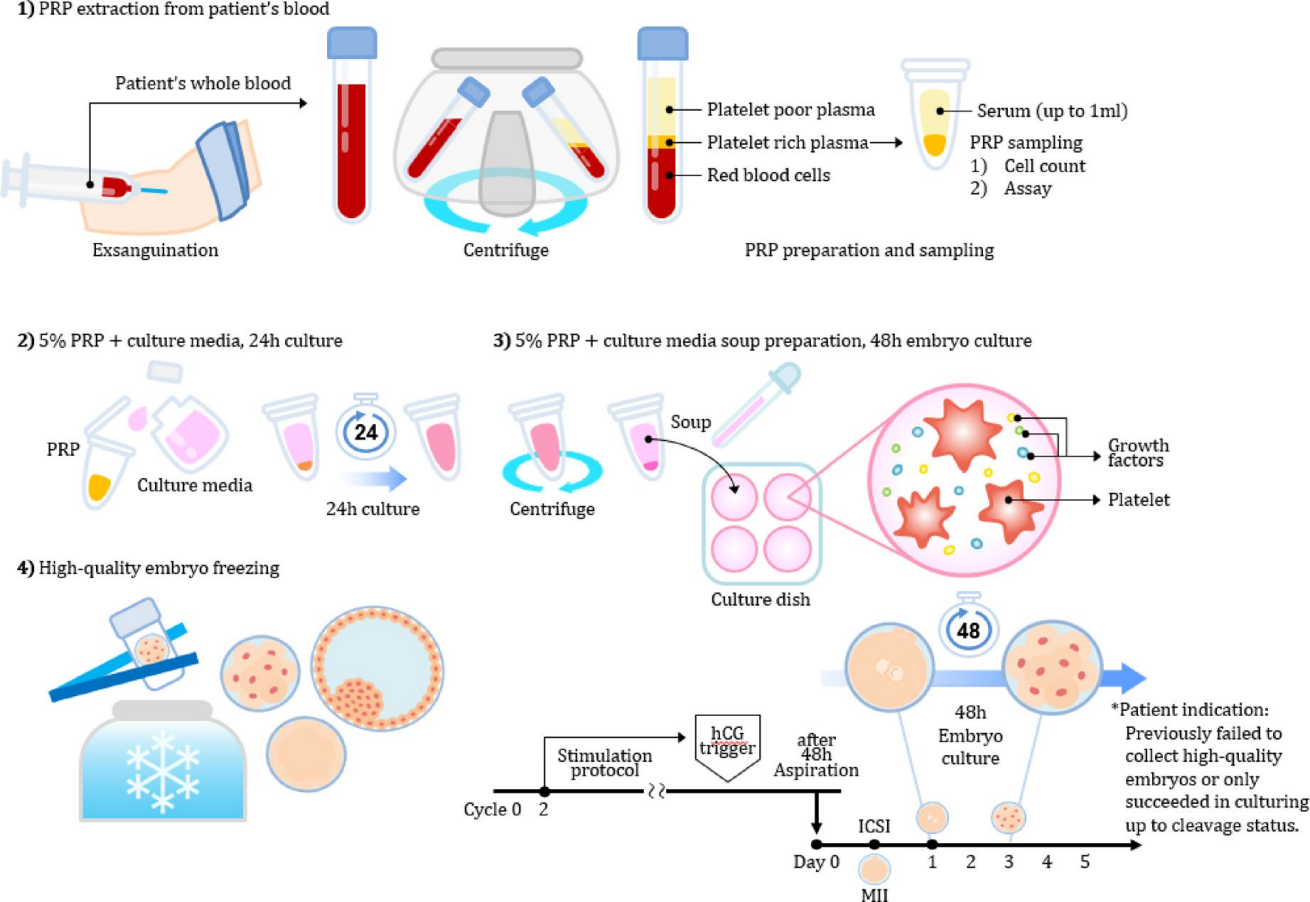
Procedure for preparing autologous PRP-added embryo culture. 14 mL of venous blood was drawn from a female participant and centrifuged at 3500 rpm for 5 min, resulting in three distinct layers: supernatant, an intermediate buffy coat layer, and red blood cells. The intermediate-layer PRP was collected carefully using a 1-mL syringe equipped with an 18-G needle and then adjusted to a final volume of 1 mL in a 5-mL tube. PRP is added to the embryo culture medium to assess its impact on embryo quality. The autologous PRP samples utilized in this study yielded a mean of 704 ± 248 × 10^3^ cells/μL (mean ± SD), which ranged from 166 to 1265 × 10^3^ cells/μL.

The procedure for preparing PRP followed the protocol outlined in our previous study^13^. An autologous PRP sample was collected from a female participant, who had no diagnosed blood diseases, on the day of oocyte pick-up. A total of 15 mL of peripheral blood was collected from the patient into a tube containing 1.5 mL of acid citrate solution as an anticoagulant. The sample was mixed thoroughly by gentle inversion to ensure proper anticoagulation. The anticoagulated blood was then processed using a commercial separation system (Ycellbio Kit; Ycellbio, Inc., Seoul, Korea). A two-step centrifugation was performed using a swing-type rotor centrifuge (Labofuge 400R, Heraeus, Germany). The first centrifugation was conducted at 3500 rpm for 5 min, immediately followed by a second centrifugation under the same conditions (3500 RPM, 5 min) depending on the buffy coat thickness and the height of the red blood cell layer. After centrifugation, the blood separated into three distinct layers: platelet-poor plasma (top), buffy coat (middle), and red blood cells (bottom). The PRP fraction was located within the buffy coat, at the interface between the plasma and RBC layers. It was carefully aspirated together with a portion of the overlying plasma using a 1-mL syringe fitted with an 18-gauge needle. Particular care was taken to avoid disturbing the RBC layer and to minimize contamination. The collected PRP was gently homogenized and adjusted to a final volume of 1 mL, comprising both the PRP and a portion of plasma. The final product was transferred into a sterile 5 mL tube and used immediately for subsequent application (Fig. 1).

### Controlled ovarian hyperstimulation, oocyte collection, fertilization, and embryo culture

Controlled ovarian hyperstimulation, oocyte collection, fertilization method, and embryo culture were performed according to published protocols^14^. Briefly, controlled ovarian stimulation was achieved using either a GnRH agonist (leuprorelin acetate 0.5 mg daily) long protocol or GnRH antagonist protocol (Orgalutran; MSD, Cetrotide; Merck Serono Ltd., Ganilever; LG Chem.). Gonadotropin dosage (Pergoveris, Gonal-F; Merck Serono Ltd., Follitrope; LG Chem., Puregon; MSD) was adjusted based on patients’ AMH levels, AFC, and previous response to stimulation. The follicular response was assessed by transvaginal ultrasonography and serum estradiol levels, and the final oocyte maturation was induced by administering hCG (Ovidrel; Merck Serono Ltd.) when at least one dominant follicle reached a size of ≥ 18 mm or when 2–3 leading follicles reached 17 mm. Oocyte retrieval was performed 36–48 h after triggering. After oocyte retrieval, oocyte–cumulus complexes (OCCs) were washed. The fertilization method, whether conventional IVF, half intracytoplasmic sperm injection (ICSI), or ICSI, was determined based on sperm quality and the reproductive history of the couple. Embryos were cultured in an embryo culture medium (Gems, Genea Biomedx Pty Ltd, Australia) under oil covering (Ovoil, Vitrolife AB, Sweden) at 37 °C in an environment containing 6% CO_2_, 5% O_2_, and 89% N_2_.

In cycles where autologous PRP was added, the prepared autologous PRP was mixed with the cleavage-stage culture medium, and embryos were incubated for 48 h. Consequently, embryos in the PRP-added cycles were cultured using the PRP-supplemented media to enhance embryo development from the initial 2PN stage to the cleavage stage.

### Embryo quality evaluation and grading

Methods for evaluating fertilization and cleavage-, morula-, and blastocyst-stage morphological grading were performed following our previous process, which was extensively detailed previously^15–18^. Briefly, after conventional IVF or ICSI procedures, 2PN confirmation was performed between 16 and 20 h post-insemination. Cleavage-stage embryos were defined as 2-cell stage or higher and assessed based on their cell division rate and morphology. High-quality cleavage-stage embryos were identified as 8-cell stage embryos with symmetric or < 10% fragmentation, whereas blastomeres were selected based on their morphology (e.g., 8-cell G2 or higher, indicating good quality). Morula-stage embryos were graded based on parameters including qualities of full and partial compaction. “Full compaction” indicated complete compaction that nearly all blastomeres were compacted, whereas “partial compaction” indicated incomplete compaction with two or three distinct blastomeres remaining incompacted. This study considered conditions indicating fully compacted embryos as indicators of high-quality embryos and partially compacted embryos as indicators of low-quality embryos. The blastocyst stage was assessed according to Gardner and Schoolcraft criteria, focusing on evaluating blastocoel expansion, inner cell mass, and trophectoderm (TE) morphology. High-quality blastocysts included expanded, mid-stage, and early-stage blastocysts graded AA, AB/BA, BB, AC/CA, or BC/CB. Low-quality blastocysts were those graded CC, regardless of developmental stage. In all stages, usable embryos were defined as surviving moderate- to high-quality embryos that were transferred or vitrified based on stage-specific morphological criteria according to the updated ESHRE/ALPHA Istanbul consensus^19^.

### Measurement of cytokines using proteome profiler human XL cytokine arrays and image analysis

Patients’ autologous PRP samples underwent analysis using the Proteome Profiler Human XL Cytokine Array Kit (R&D Systems, Minneapolis, MN, USA), which included 105 distinct capture antibodies immobilized on a nitrocellulose membrane, following the manufacturer’s guidelines. Specifically, Human XL cytokine arrays were exposed to a final volume of 1.5 mL (0.2 mL PRP + 1.3 mL diluent) of the desired culture supernatant at 4 °C for 17 h. Subsequent procedures were conducted according to the manufacturer’s instructions. Cytokine Immuno-spots array file images were analyzed using an Amersham Imager 680 digital scanner, and data were processed using the QuicK Spot Tool (Western Vision Software) and normalized against the average signal of the membrane reference spots.

### Statistical analysis

All statistical analyses were performed using JMOVI (version 2.3, JAMOVI Project, Sydney, Australia) or IBM SPSS Statistics version 25.0 (IBM Corp., Armonk, NY, USA). Continuous variables are reported as means ± standard deviations (SD) or percentages of the total. The chi-square test was used to compare qualitative variables and the T-test for quantitative variables. Significance was determined as *p* < 0.05.

## Results

### Patients’ characteristics

The study included 123 patients with infertility, comprising 175 IVF cycles in total. Among the enrolled couples, the average age of the women was 39.8 ± 4.00 years and that of men was 41.3 ± 4.47 years, with only 16 women aged ≤ 35 years. The duration of infertility was 3.97 ± 3.34 years, and the average number of previous IVF cycles was 6.38 ± 3.54. The mean AMH value was 2.13 ± 2.08 ng/mL, and the AFC was 10.6 ± 7.63. Patients with primary infertility accounted for 87.8% of the total, and the average BMI of the women was 21.9 ± 3.16 (Table 1).

**Table 1:** Baseline characteristics of patients.

Parameters	
No. of cycles	175
Patients (n)	123
Female age, (years, range)	39.8 ± 4.00 (29–49)
Male age, (years, range)	41.3 ± 4.47 (31–52)
Infertility duration, (years, range)	3.97 ± 3.34 (1–16)
Previous IVF cycles (n, range)	6.38 ± 3.54 (2–19)
Parity (n, %)	
Primary infertility	108 (87.8%)
Secondary infertility	15 (12.2%)
Female BMI, kg/m^2^ (range)	21.9 ± 3.16 (17.9–35.3)
AMH (ng/mL, range)	2.13 ± 2.08 (0.01–17.02)
Basal FSH concentration (IU/L, range)	6.19 ± 3.91 (0.86–27.81)
Basal E_2_ concentration (pg/mL, range)	64.6 ± 74.0 (5.25–493)
Basal LH concentration (IU/L, range)	4.38 ± 2.94 (0.22–15.7)
Basal Prolactin concentration (ng/mL, range)	17.1 ± 7.04 (4.07–50.0)
Basal TSH concentration (mIU/mL, range)	1.73 ± 0.818 (0.19–4.91)
Antral follicle counts (n, range)	10.6 ± 7.63 (1–50)
Cycles day (range)	15.1 ± 2.63 (9–33)

Values are presented as mean ± standard deviation (SD) or number (%).

BMI, body mass index; AMH, anti-Mullerian hormone; E_2_, estradiol; FSH, follicle-stimulating hormone; LH, luteinizing hormone; TSH, thyroid-stimulating hormone.

### Platelet concentration in PRP

To verify the platelet concentration after preparing PRP, a 150-μL sample was sent to the laboratory, and the autologous PRP samples utilized in this study yielded a mean of 704 ± 248 × 10^3^ cells/μL (mean ± SD), which ranged from 166 to 1265 × 10^3^ cells/μL. When classified according to the POSEIDON criteria, no significant differences were observed in platelet concentration among the groups (*p* = 0.350).

### Comparison of embryo development between the PRP and control groups

**Fig. 2:**
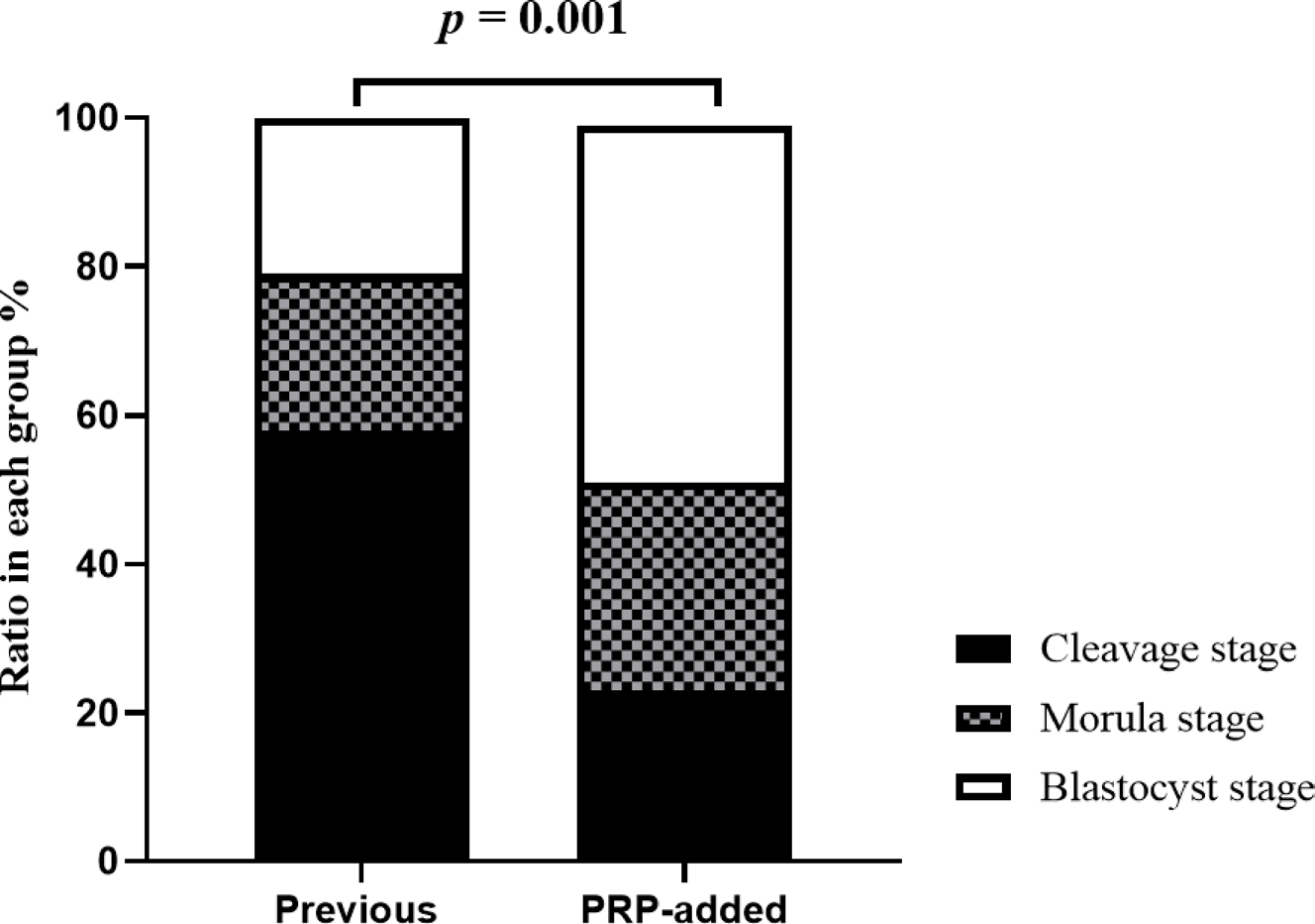
Ratio of usable embryo classified according to embryo stage between previous and PRP-added groups. The PRP-added group exhibited a higher proportion of morula and blastocyst stages and a lower proportion of cleavage stage compared to the previous group (usable embryo ratio of PRP group vs. previous group; 48.4% vs. 20.8% in blastocysts stage, *p* = 0.001; 28.0% vs. 21.4% in morula stage, *p* = 0.142; 23.6% vs. 57.9% in cleavage stage, *p* = 0.001).

**Fig. 3:**
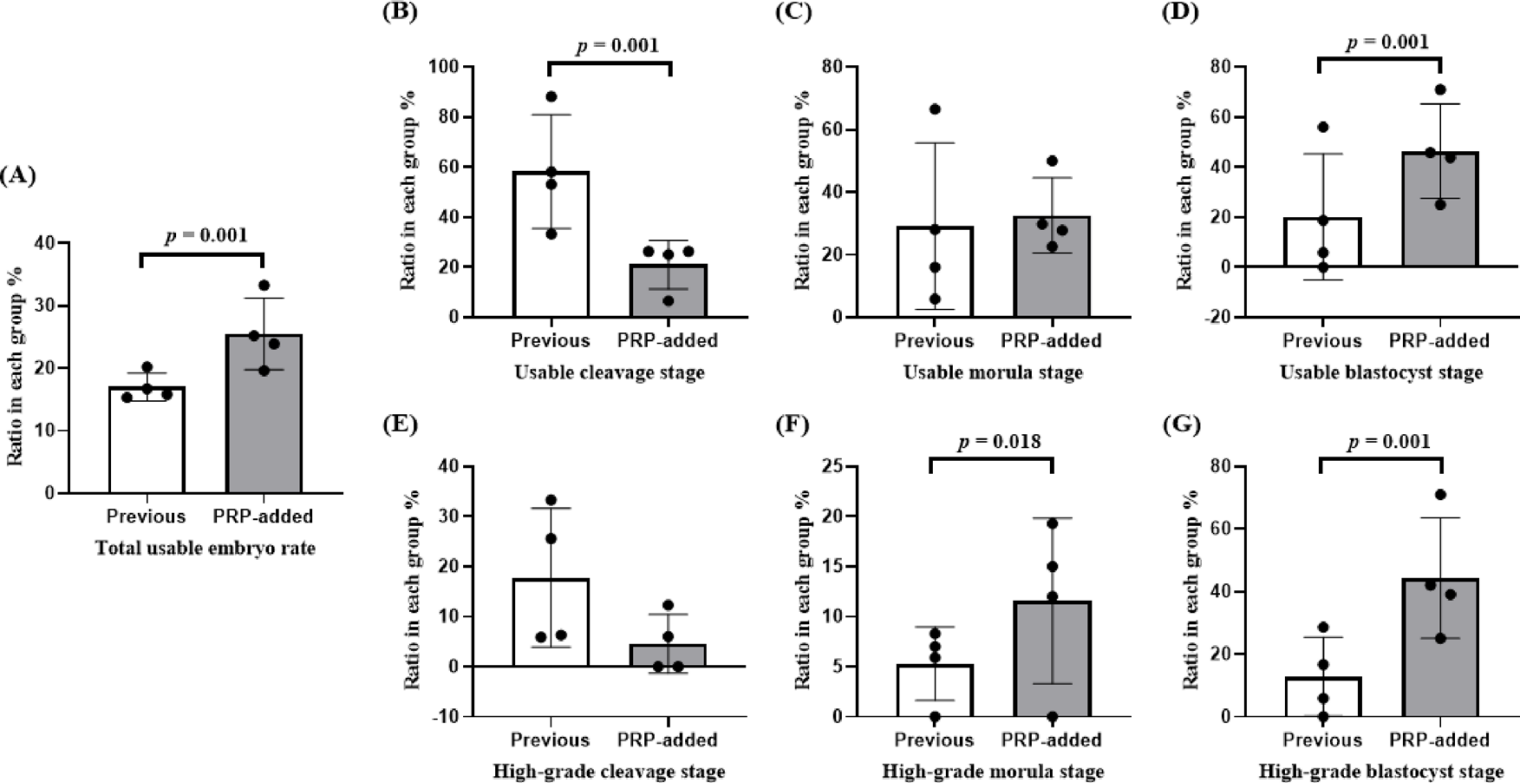
Comparison of usable embryo and high-grade embryo at each stage between previous and PRP-added groups in all study participants. (**A**) Total usable embryo rate showed higher numbers in PRP-added group (21.4% in PRP group vs. 15.8% in previous group, *p* = 0.001). (**B**–**D**) Ratio of usable embryo classified according to embryo stage. The PRP group showed a significantly lower rate at the cleavage stage and a significantly higher rate at the blastocyst stage (PRP group vs. previous group; 23.6% vs. 57.9% in cleavage stage, *p* = 0.001; 28.0% vs. 21.4% in morula stage, *p* = 0.142; 48.4% vs. 20.8% in blastocysts stage, *p* = 0.001). (**E**–**G**) Ratio of high-grade embryo classified according to embryo stage. While there was no significant difference at the cleavage stage, the ratio of high-grade embryo was lower in the PRP group, similar to the changes observed in total usable cleavages (PRP group vs. previous group; 6.7% vs. 11.9% in cleavage stage, *p* = 0.073). At the morula and blastocyst stages, the ratio of high-grade embryo was significantly higher in the PRP group (PRP group vs. previous group; 15.6% vs. 7.5% in morula stage, *p* = 0.018; 44.0% vs. 17.6% in blastocysts stage, *p* = 0.001). (High-grade criteria: Cleavage stage; ≥ 8 cells G2, Morula stage; ≥ fully compacted, Blastocyst stage; ≥ early-stage graded BC/CB).

**Fig. 4:**
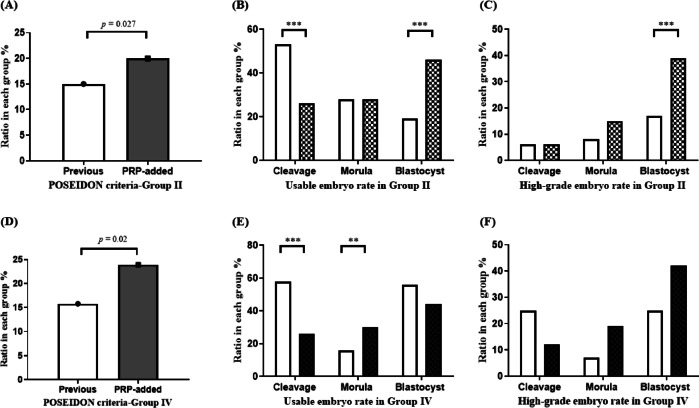
Comparison of stage-specific usable and high-grade embryo ratios between previous and PRP-added groups in groups II and IV according to the POSEIDON criteria (The shaded and pattern-filled bar graph represents the PRP-added group. ***p < 0.001, **p < 0.01). (**A**) Total usable embryo rate in POSEIDON group II showed higher numbers in PRP-added group (19.6% in the PRP-added group vs. 15.3% in the previous group, *p* = 0.027). (**B**–**C**) Usable embryo rate at the cleavage stage in POSEIDON group II was significantly lower. Usable and high-grade embryo rates according to embryo stage in POSEIDON group II were also showing significant improvements at the blastocyst stage (usable embryo rates in PRP-added group vs. previous group; 26.3% vs. 53.1% in cleavage stage, *p* = 0.001; 27.8% vs. 28.1% in morula stage, *p* = 0.959; 45.9% vs. 18.8% in blastocyst stage, *p* = 0.001; high-grade embryo rates in PRP-added group vs. previous group; 6.0% vs. 6.3% in cleavage stage, *p* = 0.942; 15.0% vs. 8.3% in morula stage, *p* = 0.126; 39.1% vs. 16.7% in blastocyst stage, *p* = 0.001). (**D**) Furthermore, comparison of total usable embryo rate in POSEIDON group IV was higher in PRP-added group (23.9% in the PRP-added group vs. 15.8% in the previous group, *p* = 0.020). (**E**) Usable embryo rates according to embryo stage in POSEIDON group IV. In the PRP group, the ratio of usable embryo was significantly lower at the cleavage stage and significantly higher at the morula stage (PRP-added group vs. previous group; 26.3% vs. 58.1% in cleavage stage, *p* = 0.001; 29.8% vs. 16.0% in morula stage, *p* = 0.013; 43.9% vs. 56.0% in blastocyst stage, *p* = 0.251). (**F**) High-grade embryo rates according to embryo stage in POSEIDON group IV did not show statistically significant differences at all stages. However, in the PRP group, there was a decreasing trend at the cleavage stage and an increasing trend at the morula and blastocyst stages (PRP-added group vs. previous group; 12.3% vs. 25.6% in cleavage stage, *p* = 0.087; 19.3% vs. 7.0% in morula stage, *p* = 0.079; 42.1% vs. 25.6% in blastocyst stage, *p* = 0.086).

The number of retrieved oocytes (10.7 ± 5.96 vs. 11.0 ± 7.06, *p* = 0.311) and fertilized embryos (6.05 ± 3.78 vs. 6.29 ± 4.39, *p* = 0.074) were not different between the PRP group and the previous group without PRP (Table 2). Table 2 also shows significant improvements in ratios of embryos at each consecutive developmental stage in PRP-added group (2-cell 99.7% vs. 95.2%, *p* = 0.001; 8-cell 48.2% vs. 28.7%, *p* = 0.001; morula 33.1% vs. 24.8%, *p* = 0.017; blastocyst 65.9% vs. 51.5%, *p* = 0.040). Regarding the distribution of usable embryos by developmental stage, the PRP group showed a significantly increased proportion of development at the morula and blastocyst stages (*p* = 0.001, Fig. 2). Furthermore, the PRP group exhibited significantly higher ratios of total usable embryos (21.4% vs. 15.8%, *p* = 0.001) and blastocyst-stage embryos (48.4% vs. 20.8%, *p* = 0.001) than the previous group. The ratio of high-grade embryos at the morula (15.6% vs. 7.5%, *p* = 0.018) and blastocyst stages (44.0% vs. 17.6%, *p* = 0.001) was also significantly higher in the PRP group than in the previous group (Fig. 3). Given the limited number of patients aged < 35 years, only patients classified as groups II and IV according to the POSEIDON criteria were included in the analysis of embryo development, where both groups showed significantly higher ratios of total usable embryos than the previous cycle without PRP (Fig. 4). In group II, a significant increase was found in the rate of usable embryos (19.6% in the PRP group vs. 15.3% in the previous group, *p* = 0.027) and high-grade embryo ratios at the blastocyst stage (39.1% in the PRP group vs. 16.7% in the previous group, *p* = 0.001). In group IV, a significant increase in the rates of usable embryos was found (23.9% in the PRP group vs. 15.8% in the previous group, *p* = 0.02). Although the high-grade embryo ratio did not show significant results at each stage in group IV, the cleavage stage tended to decrease, whereas the proportions of morula and blastocyst stages increased (PRP group vs. previous group; 12.3% vs. 25.6%, *p* = 0.087 in the cleavage stage; 19.3% vs. 7.0%, *p* = 0.079 in the morula stage; and 42.1% vs. 25.6%, *p* = 0.086 in the blastocyst stage). In addition, groups II and IV exhibited a decrease in the ratios of cleavage-stage usable and high-grade embryos (PRP group vs. previous group; usable embryo ratio, 26.3% vs. 53.1%, *p* = 0.001; high-grade embryo ratio, 6.0% vs. 6.3%, *p* = 0.942 in POSEIDON group II; usable embryo ratio, 26.3% vs. 58.1%, *p* = 0.001; high-grade embryo ratio, 12.3% vs. 25.6%, *p* = 0.087 in POSEIDON group IV).

**Table 2:** Comparisons of human embryonic development between previous and PRP-added group.

Total number of cycles (n = 175)	Previous	PRP-added	*P-*value
No. of retrieved oocytes (n)	11.0 ± 7.06	10.7 ± 5.96	0.311
Fertilized embryos (2PNs) per cycle (n)	6.29 ± 4.39	6.05 ± 3.78	0.074
No. of 2PN stage embryos (n)	1004	1050	
*Total number of 2-cell stage embryos (n, %)/2PN stage	956 (95.2%)	1047 (99.7%)	0.001
*Total number of 8-cell stage embryos (n, %)/2-cell stage	274 (28.7%)	505 (48.2%)	0.001
*Total number of M stage embryos (n, %)/8-cell stage	68 (24.8%)	167 (33.1%)	0.017
*Total number of BL stage embryos (n, %)/M stage	35 (51.5%)	110 (65.9%)	0.040

Values are presented as mean ± standard deviation (SD) or number (%).

PN, Pronucleus; M, morula; BL, blastocyst.

*The developmental rate of embryos at the specific stage.

### Comparison of the expression levels of cytokines in PRP

**Fig. 5:**
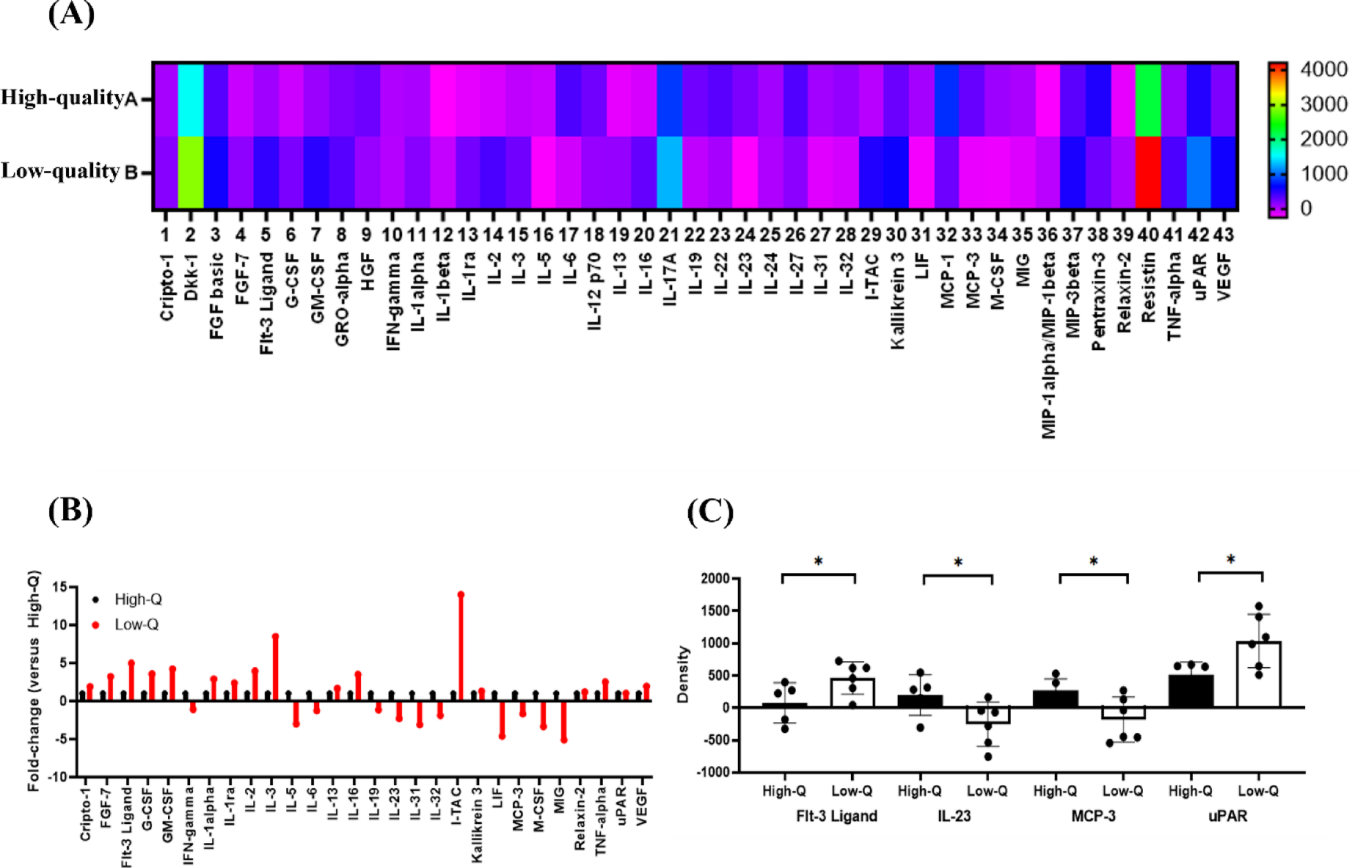
Evaluation of the expression level of proteins from autologous PRP using the proteome profiler human XL cytokine array kit. (**A**) Heatmap analysis was done to visualize signal-density measurements obtained from the cytokine array kit. Among the 105 proteins examined, 43 proteins exhibited a difference of at least 0.5-fold between the groups of successful and unsuccessful embryo development. The spots enclosed in outlined boxes were used for signal normalization. The scale bar in the heatmap represents unitless measurements, determined based on pixel density. (**B**) The presented data demonstrate significant alterations, ranging from at least 1.0- to 14-fold changes, in protein levels normalized to the array reference, indicated by the line boxes, observed between patients with high-quality embryos and those with low-quality embryos. The mean pixel-density value of the control subject was standardized to 1.0, and subsequent fold changes were computed for each protein. The data are expressed as the mean fold-change. (**C**) Among the 28 proteins tested, four proteins including FMS-like tyrosine kinase 3 (Flt-3), interleukin (IL)-23, monocyte chemoattractant protein-3 (MCP-3), and urokinase plasminogen activator receptor (uPAR) were detected. Significant differences were found in Flt-3 (28.10 in high-quality embryos vs. 498.99 in low-quality embryos), IL-23 (208.94 in high-quality embryos vs. − 405.13 in low-quality embryos), MCP-3 (304.23 in high-quality embryos vs. − 295.80 in low-quality embryos), and uPAR (472.98 in high-quality embryos vs. 1147.51 in low-quality embryos). Statistical differences were determined using paired* t*-tests (*p < 0.05). (High Q: high quality embryo; Low Q: low quality embryo).

In this study, the Human XL Cytokine Array Kit was used to identify cytokines from PRP that improve embryo development. Patients were divided into groups based on successful and unsuccessful embryo development, and the expression levels of each cytokine were compared. Of the 105 proteins tested, 43 showed at least a 50% difference in expression levels between the two groups. Of these, 28 proteins exhibited more than a 100% difference in the group that developed high-quality embryos. Significant differences were found in FMS-like tyrosine kinase 3 (Flt-3) ligand (28.10 in high-quality embryos vs. 498.99 in low-quality embryos), interleukin (IL)-23 (208.94 in high-quality embryos vs. − 405.13 in low-quality embryos), monocyte chemoattractant protein-3 (MCP-3) (304.23 in high-quality embryos vs. − 295.80 in low-quality embryos), and urokinase plasminogen activator receptor (uPAR) (472.98 in high-quality embryos vs. 1147.51 in low-quality embryos) (*p* < 0.05) (Fig. 5).

## Discussion

For the past few decades, interest in PRP for applications in diverse clinical fields such as orthopedics, ophthalmology, dental procedures, and wound management has been increasing^20–22^. These autologous platelet concentrates are also preferred in regenerative medicine owing to their high safety and efficacy, convenient preparation process, lack of immunogenicity, and minimal potential risks^2,5,23^. From the perspective of reproductive medicine, emerging evidence presents potential benefits of intrauterine or intraovarian administration of PRP for improving ART outcomes in women with infertility, thin endometrium, recurrent implantation failure, or poor ovarian reserve^5,24,25^.

The precise mechanism of PRP therapy has not been fully clarified so far; however, the involvement of pathways such as the induction of cell proliferation and migration, chemotaxis, cell regeneration, extracellular matrix synthesis, remodeling, angiogenesis, and epithelialization has been proposed^2,5^. These pathways are believed to be mediated by various growth factors and cytokines contained in the α-granules of platelets. Growth factors are present in α-granules, such as the platelet-derived growth factor, transforming growth factor-β, epidermal growth factor, vascular endothelial growth factor, fibroblast growth factors, insulin-like growth factor, and hepatocyte growth factor^2,5^. These growth factors well-known to be included in PRP may participate in endometrial and ovarian regeneration^2,5^. In addition, some of the abovementioned growth factors have shown favorable outcomes as supplements in human IVF culture media, such as increased pregnancy rates alongside accelerated development, improved quality, higher blastocyst formation rates, increased cell numbers, earlier hatching, and enhanced implantation rates^6^.

Based on the abovementioned findings, several studies have investigated whether PRP could substitute for other serum preparations, such as human serum albumin or fetal bovine serum, which is often used as a supplement in cell cultures^11,26–30^. Studies have also shown that PRP may improve oocyte maturation, fertilization, and embryo development in animal models^9,10,31^ beyond endometrial and ovarian regeneration. Moreover, the addition of PRP to the in vitro culture media of isolated human pre-antral follicles could enhance follicle survival and growth^11,30^. However, no studies have examined whether PRP supplemented in embryo culture media can improve human embryo development and quality. Therefore, this study explored whether supplementing culture media with PRP in patients undergoing IVF who previously had poor embryo outcomes demonstrates favorable effects on embryo development and a number of vitrified embryos.

In this study, the composition of the embryo culture media in the previous and PRP groups was identical, except for the addition of PRP in the latter group. When comparing embryonic development between previous cycles without PRP and a present cycle with PRP supplementation in the same patients, the total usable embryo rate in the PRP-added cycles increased. High-grade embryos in morula and blastocyst stages showed a significant increase. Furthermore, in groups II and IV classified by the POSEIDON criteria, the ratio of the total usable embryos significantly increased in the PRP group. Overall, a trend of decreased cleavage-stage embryos and increased morula- and blastocyst-stage embryos was noted. These results indicate that the growth factors and cytokines contained in PRP may support the preimplantation embryo development.

Furthermore, to compare the cytokines present in PRP added to the embryo culture media, the Human XL Cytokine Array kit was utilized. Patients were categorized into high- and low-quality embryo groups, and the expression levels of cytokines in PRP were compared. Among them, expression levels of Flt-3 ligand, IL-23, MCP-3, and uPAR were significantly different. uPAR is the receptor that pro-uPA (urokinase plasminogen activator) binds to. uPA, activated from pro-uPA by uPAR, converts plasminogen to plasmin. Plasmin activates matrix metalloproteinases (MMPs) and growth factors, degrading the ECM and promoting invasion, which is crucial for embryo implantation^32^. The interaction between the embryo and its surrounding environment is crucial for early embryo development, and MMPs may play a role in this process^33,34^. The plasminogen activator system can also enhance embryo development. In mouse embryos, the expression levels of uPA and uPAR increase during blastocyst formation before implantation^35^. In human preimplantation embryos, uPA expression is higher at the blastocyst stage than at the 2–4 cell stage^36^. Thus, adding uPA and plasminogen to the culture media of mice embryos promotes development to the blastocyst stage compared with the control group^35^.

Flt-3 ligand binds to Flt-3, the tyrosine kinase receptor, and activates intracellular signaling and promoting cell proliferation and survival^37^. Although it primarily regulates the proliferation and differentiation of hematopoietic progenitor cells^38^, Flt-3 ligand is expressed in the oviduct and uterus of pregnant mice during preimplantation. A study reported that treating early embryonic stages with Flt-3 ligand dose-dependently enhances development to morula and blastocyst stages^39^. Moreover, adding uPAR and Flt-3 ligand to the embryo culture media could be beneficial for embryo development. However, in the present study, PRP with higher expression levels of these cytokines was associated with low-quality embryos. This may be attributed to excessive amounts of cytokines than necessary, which may have been detrimental to embryo development, although the optimal PRP concentration has not been established and the appropriate levels of each cytokine remain unknown.

The balance of Th1/Th2 and Th17/Treg type cytokines between the maternal uterus and the fetoplacental unit is crucial during embryo implantation^40,41^. When considering IL-1β, one of the inflammatory cytokines that regulate the balance between Th1 and Th2 cells, its levels gradually increase, reaching their peak at the blastocyst stage during embryonic development^42,43^. Moreover, studies have reported that the external supplementation of IL-1β in mouse embryo culture media enhances blastocyst quality, increases hatching rates, and accelerates hatching speed^44,45^. Conversely, excessively high IL-1β levels has been associated with an increased risk of pregnancy complications, such as miscarriage, preeclampsia, and preterm birth^46^. In conclusion, IL-1β is essential for embryo development and the early implantation process, but its expression must be finely balanced to ensure a successful pregnancy^42–46^. This highlights the dual role of cytokines in embryonic development. Secreted cytokines act as key mediators of communication between the female reproductive system and the embryo, exerting not only beneficial nutritional effects but also potentially adverse influences on embryonic development.

MCP-3 is a proinflammatory chemokine belonging to the CC chemokine family (also known as CCL7) and is one of the Th1-type cytokines that can hinder embryo implantation. In mouse studies, MCP-3 was expressed in the endometrium and embryos during preimplantation, its expression decreased as embryo development progressed, and it was completely absent in the post-implantation stage^40^. IL-23 is a cytokine that stimulates the differentiation of Th17 cells, which secrete the proinflammatory cytokine IL-17. Studies have shown increased IL-23 expression in decidual cells and peripheral blood of patients with recurrent pregnancy loss^41^. However, research on the role of IL-23 in embryo development is limited.

**Fig. 6:**
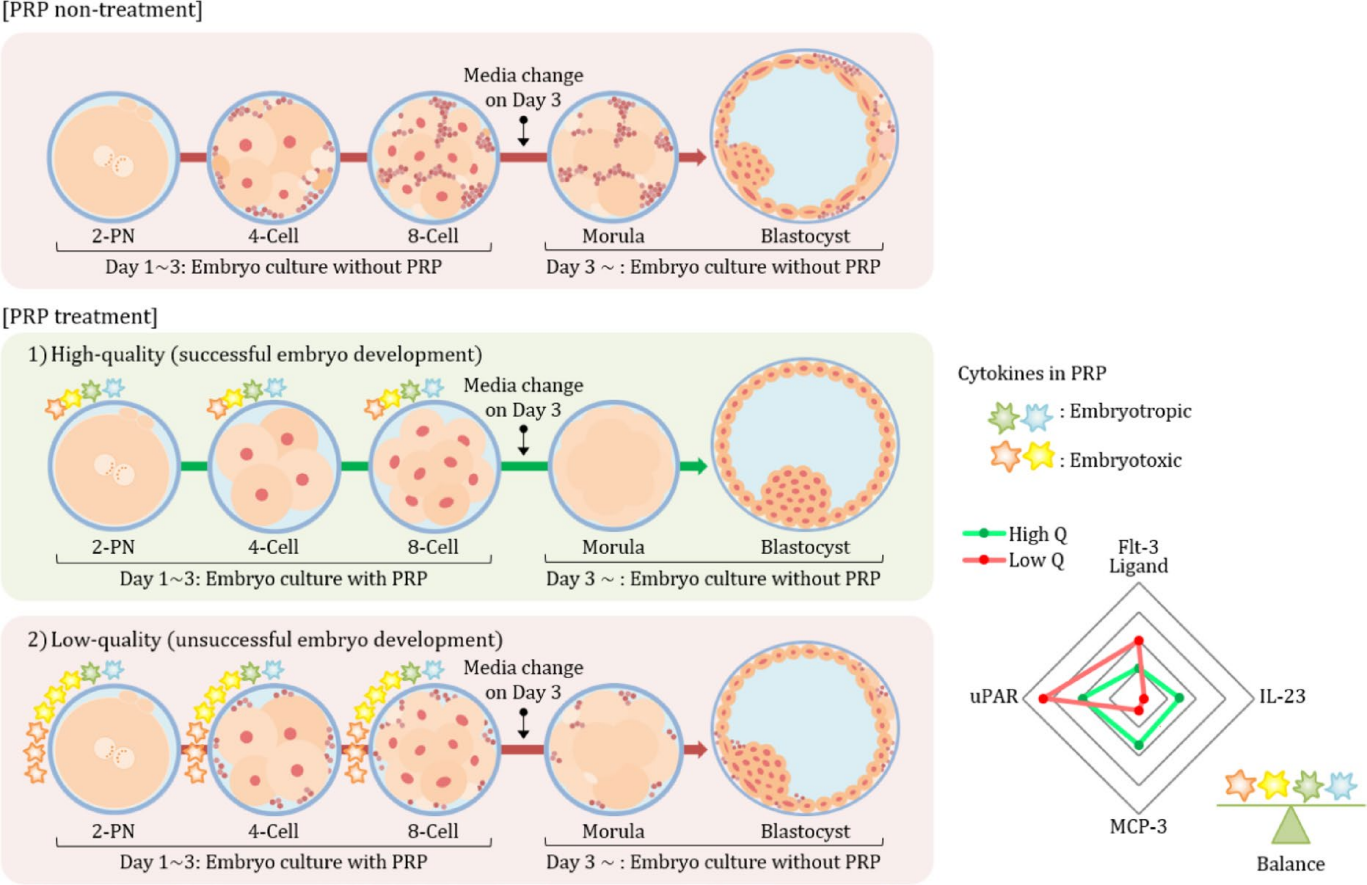
Overview of the use of autologous PRP in embryo culture to obtain high-quality embryos. The presence of both anti-inflammatory and proinflammatory cytokines in appropriate concentrations is crucial for optimal embryo development. The differences in cytokines present in PRP added to the embryo culture media of high- and low-quality embryos may alter immune-inflammatory factors, thereby influencing cell-developmental interactions. The addition of PRP to the embryo culture medium in patients with poor prognoses may improve embryonic development. Further analysis of the cytokines contained in PRP and their effects on embryo development will enhance our understanding of the cellular mechanisms underlying embryonic development. (High Q: high quality embryo; Low Q: low quality embryo).

Notwithstanding MCP-3 and IL-23 are proinflammatory cytokines and when at high expression levels are expected to negatively affect embryo development, the results of this study showed that higher expression levels of these cytokines in PRP are associated with high-quality embryo development. Although the exact mechanisms of MCP-3 and IL-23 in embryo development are not fully understood, not only anti-inflammatory cytokines but also proinflammatory cytokines are required in appropriate concentrations for optimal embryo development^47^. This implies that when anti-inflammatory cytokines and proinflammatory cytokines achieve a proper balance in the immune response, they are thought to positively influence the microenvironment surrounding the embryo and embryonic development. In addition, the interaction with other growth factors present in PRP might have contributed positively to embryo development. As mentioned above, the optimal PRP concentration and appropriate cytokine levels showing significant results in this study have not been established, indicating a need for further research in this area. Figure 6 schematically illustrates this mechanism.

To the best of our knowledge, this is the first study that investigated whether the addition of PRP to the culture media during human embryo culture in IVF could positively affect embryonic development. PRP commonly used in reproductive medicine is usually obtained from the patient’s blood, making it safe with no immunogenicity or potential risks, and it is relatively affordable. Although various studies have demonstrated the beneficial effects of PRP, the lack of standardized protocols for PRP preparation results in different protocols used, and no consensus on the appropriate PRP concentration has been established in each study. Thus, obtaining consistent results and comparing findings across studies is difficult. This study is limited by the fact that concentrations of white blood cells and red blood cells in the PRP were not assessed, which may affect the characteristics of PRP. Additionally, the limitations of this study include the small size and potential bias and limitations inherent to its retrospective design. Furthermore, the absence of a placebo control group and analyses of pregnancy outcomes makes it challenging to determine a precise causal relationship between PRP supplementation and embryo development. To clarify the effects of PRP in embryo culture, further large-scale human randomized controlled trials are necessary.

## Data Availability

The data used and analyzed during the current study are available from the corresponding author on reasonable request.
